# Evidence for Reductions in Physical and Chemical Plant Defense Traits in Island Flora

**DOI:** 10.3390/plants13071026

**Published:** 2024-04-03

**Authors:** Micah G. Freedman, Randall W. Long, Santiago R. Ramírez, Sharon Y. Strauss

**Affiliations:** 1 Center for Population Biology, University of California, Davis, CA 95616, USA; 2 Department of Evolution and Ecology, University of California, Davis, CA 95616, USA; 3 Department of Biology, Lewis & Clark College, Portland, OR 97219, USA

**Keywords:** islands, plant defense, marginal spines, specific leaf area, cyanogenic glycosides, *Stachys*, terpenes

## Abstract

Reduced defense against large herbivores has been suggested to be part of the “island syndrome” in plants. However, empirical evidence for this pattern is mixed. In this paper, we present two studies that compare putative physical and chemical defense traits from plants on the California Channel Islands and nearby mainland based on sampling of both field and common garden plants. In the first study, we focus on five pairs of woody shrubs from three island and three mainland locations and find evidence for increased leaf area, decreased marginal leaf spines, and decreased concentrations of cyanogenic glycosides in island plants. We observed similar increases in leaf area and decreases in defense traits when comparing island and mainland genotypes grown together in botanic gardens, suggesting that trait differences are not solely driven by abiotic differences between island and mainland sites. In the second study, we conducted a common garden experiment with a perennial herb—*Stachys bullata* (Lamiaceae)—collected from two island and four mainland locations. Compared to their mainland relatives, island genotypes show highly reduced glandular trichomes and a nearly 100-fold reduction in mono- and sesquiterpene compounds from leaf surfaces. Island genotypes also had significantly higher specific leaf area, somewhat lower rates of gas exchange, and greater aboveground biomass than mainland genotypes across two years of study, potentially reflecting a broader shift in growth habit. Together, our results provide evidence for reduced expression of putative defense traits in island plants, though these results may reflect adaptation to both biotic (i.e., the historical absence of large herbivores) and climatic conditions on islands.

## 1. Introduction

Plant defenses against herbivory are thought to be energetically costly due to allocational tradeoffs with plant growth [1,2], leading to predictions that constitutive defenses should be proportional to the risk of attack by herbivores. One approach for understanding the evolution of plant defense traits involves using naturally occurring gradients of herbivory intensity to test for concomitant variation in plant defenses, either within (e.g., [3,4]) or across species (e.g., [5,6]). Examples of this approach include comparative studies that find reductions in defensive traits in plants at higher latitudes [7] and higher elevations [8], concordant with the idea that herbivory is more intense in the tropics and at lower elevations.

One commonly studied contrast in herbivory intensity is between plants from oceanic islands and their mainland relatives. These contrasts are most informative when islands are missing entire assemblages of herbivores—particularly large mammalian herbivores that are poor overwater dispersers [9]—and their constituent floras have evolved in isolation for extended periods. Reduced defenses are often considered to be part of the “island syndrome” in plants, which also involves reductions in dispersal ability, increased woodiness, and increased reliance on clonal reproduction [10,11,12,13]. Numerous studies have used either conspecific or congeneric comparisons of island and mainland taxa and found reduced expression of putative defense traits on islands, including reductions in marginal leaf spines [14,15], prickles [16], divaricate branching [17], root alkaloids [18], and leaf tannins [19]. Many studies and reports have also noted that plants from oceanic islands are highly palatable to non-native mammalian herbivores [14,20,21,22,23], which has also been used as evidence of reduced defensive traits.

A recent meta-analysis of studies comparing island and mainland plants found evidence for increased mammalian herbivore damage in island compared to mainland plants, supporting the idea that island plants may be more susceptible to mammalian herbivores [24]. However, this meta-analysis did not find consistent support for reductions in plant defensive traits on islands across a set of 173 island/mainland comparisons, and subsequent comparative studies have also failed to support the idea of reduced defensive traits in island taxa [25]. Thus, the degree to which island plants show reduced defensive traits seems highly variable. Reasons for a lack of reduced defensive trait expression in island plants are numerous (see [26]) but might include (i) recent introduction of non-native mammalian herbivores that favor increased defensive traits, either through phenotypic plasticity or directional selection; (ii) an extended history of coevolution with native large herbivores that are no longer present (e.g., [27]); (iii) selection by invertebrate herbivores on islands; (iv) ongoing gene flow from mainland populations that limits the degree of differentiation in island populations.

Even in instances where studies do find evidence for reductions in plant defensive traits on islands, methodological drawbacks may limit the scope of these conclusions. First, many studies only include comparisons from a single conspecific or congeneric pair, and likewise, measurements may only contrast a single island and a single mainland site. Thus, it is often unclear whether trait divergence between islands and mainland plants is the product of adaptive divergence or idiosyncrasy. Second, few studies have attempted to measure plant traits from island and mainland genotypes grown under common environmental conditions (but see [28,29]), thereby raising the possibility that trait divergence is the result of differences in the abiotic environment between island and mainland sampling locations. Third, traits that are assumed to be defenses against herbivores may have alternative functions. For example, studies comparing island and mainland locations sometimes consider traits such as leaf area, leaf thickness, and condensed tannin concentrations as adaptations to herbivory, though these traits also have roles in primary metabolism and plant growth [30,31]. Finally, and related to the third point, relatively few studies verify the importance of putative defensive traits using experiments with herbivores (but see [14,18,21,32,33]).

**Figure 1:**
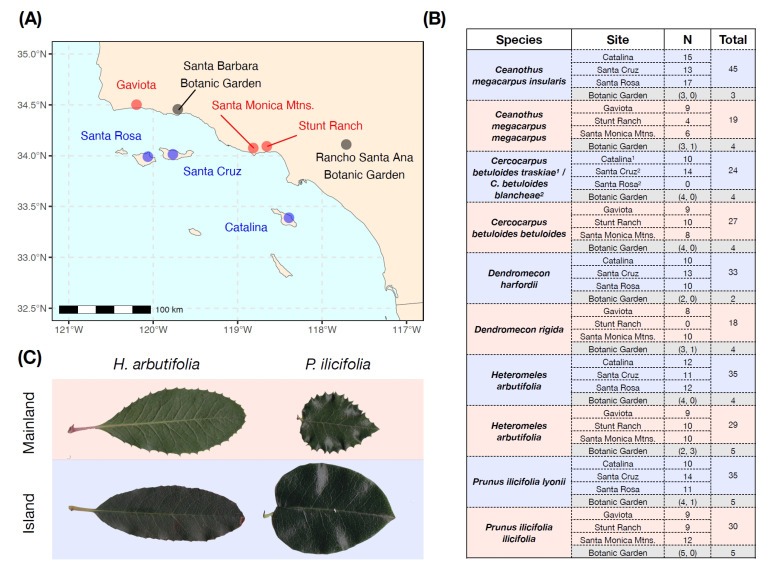
(**A**) Map of sampling locations for field and botanic garden leaf collection of chaparral shrubs. Island locations are shown in blue, mainland locations are in red, and botanic garden locations are in grey. (**B**) Table showing the number of plants sampled across each combination of species *x* site. All species were sampled from all sites, with the exception of *C. betuloides* from Santa Rosa Island. Parenthetical values refer to sampling from Rancho Santa Ana Botanic Garden and the Santa Barbara Botanic Garden, respectively. (**C**) Examples of leaves from island and mainland populations of *Heteromeles arbutifolia* and *Prunus ilicifolia*. Note reductions in marginal spines in both species.

**Figure 2:**
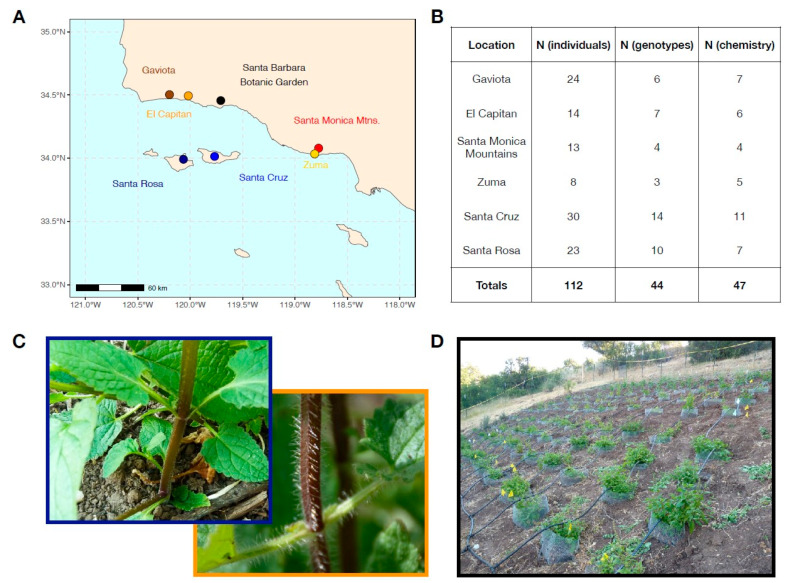
(**A**) Map of collection locations for *Stachys bullata* grown in a common garden experiment. The common garden was located at the Santa Barbara Botanic Garden (black dot). (**B**) Table showing the number of plants from each population grown in the common garden. Genotypes refer to the number of rhizomes originally propagated from discrete plant patches collected from each location. These plants were then separated to create clones within most genotypes. N (chemistry) refers to the number of plants whose leaf tissue was analyzed to determine surface chemistry using GC-MS. (**C**) Example of stem trichome density in *S. bullata* from Santa Rosa Island (**top left**) and El Capitan (**bottom right**). (**D**) Layout of the common garden plot. Photo taken in April 2016, approximately one month after transplanting.

In this paper, we present two studies comparing plant populations from the California Channel Islands and nearby mainland locations. In the first study, we used five taxonomic pairs of woody chaparral shrubs sampled across three island and three mainland sites (Figure 1), as well as two mainland botanic garden locations, to test for divergence in leaf area, specific leaf area, marginal leaf spinescence, and concentrations of cyanogenic glycosides. In the second study, we collected 44 genotypes of California hedgenettle (*Stachys bullata*) from two island and four mainland locations (Figure 2) and grew them for two years in a mainland common garden to measure plant chemistry and growth-related traits. 

## 2. Results

### 2.1. Chaparral Shrubs–Field Sampling

**Figure 3:**
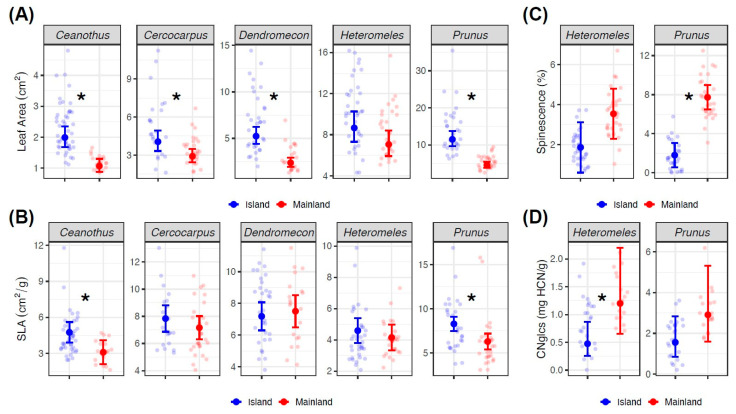
Trait values for each species across island and mainland locations, based on field sampling. Model-estimated marginal means and 95% confidence intervals are shown with solid points and lines. Each pale dot corresponds to a single plant-level mean, which is itself the mean of leaf traits from branches in the upper and lower canopy. Measured leaf traits were (**A**) leaf area, (**B**) specific leaf area, (**C**) marginal leaf spinescence (*Heteromeles* and *Prunus* only), and (**D**) concentrations of cyanogenic glycosides (*Heteromeles* and *Prunus* only). Asterisks correspond to significant (*p* < 0.05) differences between island and mainland plants within each species *x* trait combination.

We found evidence for increased leaf area and specific leaf area and decreased marginal spines and cyanogenic glycosides for island compared to mainland plants sampled in situ. Leaf area was significantly higher on islands for four of the five sampled taxa (Table 1, Figure 3A and Figure S7A). Specific leaf area (SLA) was significantly higher for two species on the islands (Table 1, Figure 3B and Figure S7B). Overall, leaves sampled from the upper canopy were significantly smaller than leaves sampled from the lower canopy (t = −2.877, p = 0.004). SLA was also lower for leaves from the upper canopy (t = −6.694, *p* < 0.001), and SLA was higher for plants growing on north-facing (t = 3.369, *p* < 0.001) and west-facing aspects (t = 3.110, *p* = 0.002).

Among traits with clearer links to defense against herbivores, we found significantly reduced marginal leaf spines in field-sampled plants for island *Prunus* and marginally reduced leaf spines in island *Heteromeles* (Table 1, Figure 3C and Figure S10). Marginal spines were less prevalent on leaves from the upper canopy (Figure S9), and spinescence heteroblasty was more pronounced in mainland plants, with larger differences in marginal spines between upper and lower canopy positions than on islands (t = 2.689, *p* = 0.008) (Figure S9). Island genotypes also had significantly reduced levels of cyanogenic glycosides (CNglcs) for *Heteromeles* and non-significant but reduced levels for *Prunus* (Table 1, Figure 3D and Figure S7D). Overall, younger leaf tissue contained significantly higher concentrations of CNglcs (t = 7.689, *p* < 0.001) regardless of location, and the magnitude of the island/mainland difference in CNglcs was more pronounced for older leaf tissue than for younger leaf tissue (t = 3.532, *p* < 0.001) (Figure S10).

We did not find any significant differences for any measured traits inside versus outside of herbivore exclosures on Catalina Island (Table S2). 

### 2.2. Chaparral Shrubs–Botanic Garden Sampling

Botanic garden plants showed a similar increase in leaf size for island genotypes, though only *Prunus* was significantly larger from islands (Table 2) (Figure S8A). SLA was not measured for botanic garden plants. Island genotypes grown in botanic gardens also showed lower spinescence, with significantly reduced marginal spines in island *Prunus* but not in *Heteromeles* (Table 2, Figure S8B). As with field-sampled plants, spinescence heteroblasty was less pronounced in island compared to mainland genotypes (t = 3.187, *p* = 0.006). CNglcs were marginally reduced in island compared to mainland genotypes of both *Prunus* and *Heteromeles* grown in botanic gardens (Table 2, Figure S8C).

**Figure 4:**
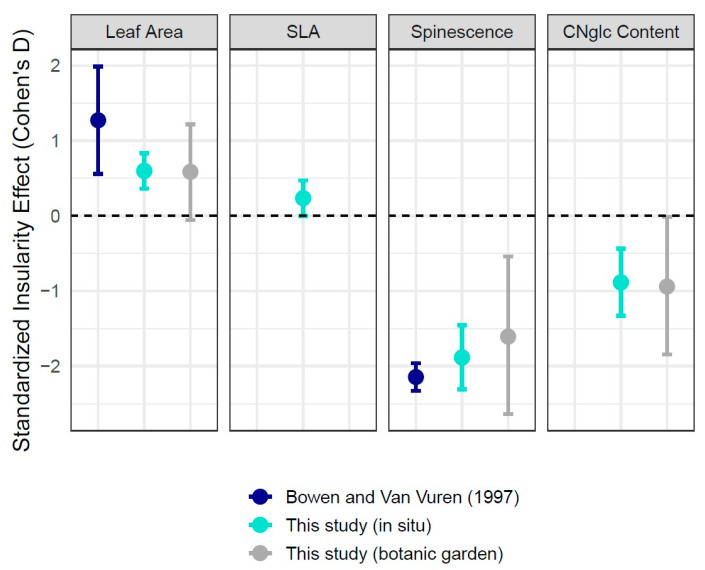
Effect sizes (Cohen’s D) and 95% confidence intervals for each island/mainland contrast across traits. Estimates are separated and colored based on the comparison. Positive values in the first two panels correspond to increased leaf area and specific leaf area for island plants. Note that SLA was not measured for botanic garden plants. Negative values in the second two panels correspond to reduced leaf spinescence and CNglc concentration in island plants [14].

Overall insularity effect sizes were comparable between our field and botanic garden sampling but lower than estimates from an earlier study (Bowen and Van Vuren [1997]) in the same system [14] (Figure 4). The strongest observed differences between island and mainland locations were for marginal spinescence, where overall insularity effect sizes ranged from −1.61 (botanic garden plants) to −1.88 (field plants) to −2.14 (Bowen and Van Vuren). 

### 2.3. Stachys Common Garden

**Figure 5:**
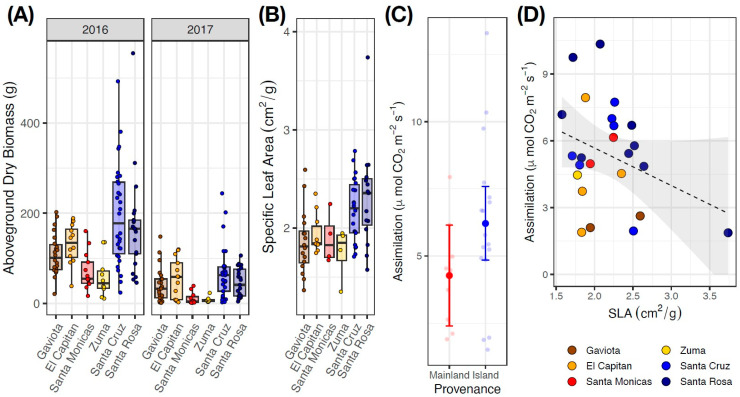
(**A**) Aboveground biomass across 2016 and 2017 for *S. bullata* populations grown at the SBBG common garden. (**B**) Specific leaf area for the same populations in 2017. (**C**) Net carbon assimilation (A_net_) measured for a subset of 26 *S. bullata* plants and separated based on provenance (island versus mainland). Island plants had marginally higher rates of A_net_. (**D**) Carbon assimilation and specific leaf area were significantly negatively correlated across the 26 sampled plants.

Of the 112 plants originally transplanted in 2016, 108 survived through the first year, and 103 survived through the second year. Plants had significantly higher biomass in 2016 when they received supplemental water (t = 12.094, *p* < 0.001). Overall, island genotypes grew significantly larger than their mainland relatives (t = 3.241, *p* = 0.048) (Figure 5A); the absolute difference in biomass across years was identical, with island-origin plants supporting an average of 54.9 g of additional biomass in each year (Figure 5A). Island plants had significantly higher SLA than their mainland relatives (t = 3.073, *p* = 0.042) (Figure 5B). Island plants generally had higher rates of gas exchange than mainland plants (Figure 5C), although this difference was not significant (t = 1.717, *p* = 0.099). Plants with higher values of SLA had lower rates of carbon assimilation (t = −2.389, *p* = 0.026) (Figure 5D).

**Figure 6:**
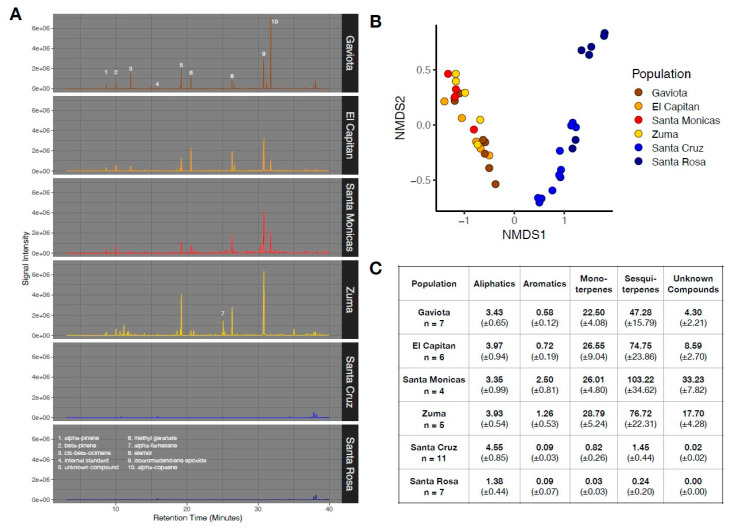
(**A**) Representative chromatograms for *S. bullata* from each of the six collection locations. Tentative identity of numbered peaks is shown in the bottom panel. (**B**) NMDS plot with samples grouped based on collection location. (**C**) Average concentration (in tetralin equivalents) for major compound classes detected in samples. Values represent mean ± standard errors.

Consistent with observations from *Stachys* growing in situ, island and mainland genotypes sampled from the common garden had markedly different chemical compositions, both in terms of absolute abundance and the presence/absence of compounds (Figure 6B) (PERMANOVA: F_site_ = 38.71, *p* < 0.001). The most pronounced chemical difference between island and mainland genotypes was for mono- and sesquiterpenes, with island genotypes showing an approximately 100-fold reduction in the abundance of these compounds (Figure 6C). Santa Cruz Island genotypes did not differ in leaf chemistry based on whether they were grown on Santa Cruz Island versus the mainland (Figure S11).

## 3. Discussion

We found general support for divergence in plant traits between islands and mainland sites, including a reduction in putative plant defense traits (marginal spines, cyanogenic glycosides, terpenes) and an increase in traits associated with growth in island plants (leaf area, specific leaf area) [30]. These results were consistent across both field-sampled and common garden plants and matched the predictions of the island syndrome. However, the magnitude of insularity effects varied widely across our sampled taxa, and we did not directly assess whether the traits we measured actually deterred mammalian herbivory. Furthermore, our results reflect some degree of ascertainment bias: the shrub species that we measured were already noted to have reduced levels of putative defenses in a previous study [14], and *Stachys bullata* was chosen for study specifically because of its pronounced phenotypic differentiation between island and mainland locations [34]. Thus, although we find evidence for reduced putative defenses in island flora, our results do not invalidate recent analyses that challenge the generality of reduced defenses as part of the island syndrome (e.g., [24]).

One of the clearest traits that showed reduced expression in island taxa was marginal leaf spinescence (Figure 1B), consistent with two previous studies of chaparral shrubs from the Channel Islands [14,33]. Spinescence in the broad sense (e.g., prickles, thorns, and leaf spines) has been suggested as a defensive adaptation for deterring mammalian herbivores [35]; spinescence may also have ecophysiological functions (e.g., [36]), although the physiological role of leaf margin morphology (including marginal spines) has rarely been evaluated (but see [37]). In a recent survey of spinescence in island flora, Barton et al. [38] highlighted many examples of island taxa that remain spinescent, potentially due to past selection by now-extinct megafauna. By contrast, with the exception of the pygmy mammoth (which has been extinct since the Late Pleistocene) and the very recent introduction of grazing animals, the Channel Islands flora is thought to have evolved without any large mammalian herbivores present. 

Consistent with previous research on spinescence across plant development, we found support for a pattern of spinescence heteroblasty, with leaves lower in the canopy showing greater spinescence (e.g., [39]). This pattern was more pronounced for mainland plants, mirroring the results of Burns (2014) [16], who also found a weaker vertical gradient in leaf spinescence within *Drypetes deplanchei* from Lord Howe Island compared to mainland Australia. Taken together, our results suggest that the reduced marginal leaf spines in the Channel Islands flora may reflect reduced selection by mammalian herbivores.

We also found evidence for reduced cyanogenic glycosides (CNglcs) on islands in the two genera that we surveyed (*Prunus* and *Heteromeles*), although the magnitude of this reduction was less pronounced than for marginal spinescence. Leaf CNglc content was lower for island compared to mainland plants grown in botanic gardens, suggesting a genetic basis for this reduction. CNglcs are acutely toxic to many vertebrate herbivores and are generally thought to have evolved as defenses against herbivores ([40] and references therein). Many other studies have found no difference [32] or even increased chemical defenses for island plants [41], and at least one study found evidence for increased levels of CNglcs in relict island populations of *Prunus lusitanica* [42]. One potential explanation for differences in CNglc content relates to climate and freeze susceptibility; because CNglcs are spontaneously converted to HCN with freeze/thaw cycles, CNglc production may be lower in evergreen plants subject to freezing temperatures (e.g., [43]). However, our island sites showed *less* temperature seasonality than mainland sites (Figures S3–S5), which suggests that climatic differences between sites are unlikely to be the reason for reduced CNglcs in island *Heteromeles* and *Prunus*. Finally, we also found strong evidence for ontogenetic decreases in CNglc concentrations in older leaf tissue, a pattern previously shown in *Heteromeles* [44] and other cyanogenic species (e.g., [45]) and concordant with optimal plant defense theory. Thus, our observed reductions in CNglc content are consistent with relaxed selection from mammalian herbivores in the Channel Islands.

Although we did not directly assess the efficacy of putative leaf defenses against herbivores, two studies using plants from the Channel Islands have shown that mammalian herbivores perceive differences between island and mainland plants. Bowen and Van Vuren (1997) [14] showed that sheep preferentially consumed leaf tissue from plants collected on Santa Cruz Island compared to a mainland location, and Salladay and Ramírez (2018) [33] likewise showed the same pattern with goats and plant tissue from Santa Catalina Island. Thus, the reductions in spinescence and CNglcs that we measured (or potentially other correlated traits) seem to be reasonable proxies for increased palatability to mammalian herbivores. Because we sampled the same taxa as Bowen and Van Vuren [14] (and at the same time of year), we can directly compare our data on leaf area and marginal spines from Santa Cruz Island to theirs. Insularity effects are in the same direction (Figure 4), although the magnitude of island/mainland differences reported in Bowen and Van Vuren is larger. 

In our common garden study of *S. bullata*, we found unambiguous evidence for reductions in leaf chemical compounds from island genotypes. These patterns were most pronounced for monoterpenes and sesquiterpenes. Because of the varied ecological functions of terpenes, it is difficult to ascribe their loss in island *S. bullata* strictly to the absence of vertebrate herbivores, though their reduction is certainly consistent with strong divergent selection between island and mainland environments. The reduction in leaf secondary compounds was also accompanied by a strong reduction in leaf and stem trichomes (Figure 2C), although we did not formally quantify their abundance.

In a common garden, island *S. bullata* genotypes had significantly thinner and larger leaves, were taller, and had woodier and more upright stems with increased branching on terminal inflorescences (Figure S12). These responses are consistent with evidence for increased woodiness among island flora [13,46]. The smaller stature of mainland *S. bullata* is reminiscent of a recent study that found that *Plectritis congesta* populations from islands without deer grow to be 2.6 times taller than populations from nearby islands with deer present [28], and with other studies showing that browsing selects for shorter stem height in *Lythrum salicaria* [47]. In addition to having higher SLA (Figure 5B) and accumulating more aboveground biomass than their mainland relatives, island *S. bullata* also had marginally higher rates of carbon assimilation (Figure 5C); however, overall rates of carbon assimilation were negatively correlated with SLA (Figure 5D). The observed negative correlation between SLA and photosynthetic capacity highlights that predicted leaf-level tradeoffs may not be as strong within species as they are at higher taxonomic levels [48]; this result is also consistent with a recent analysis of Macaronesian island flora [49], which hypothesized that reduced temperature seasonality on islands (see Figures S3–S5) drives increases in leaf size (as in [50]) but concomitant decreases in photosynthetic rates. 

An intriguing parallel to the reduced aromaticity and increased stature of island *Stachys* can be seen in the related Hawaiian mint *Haplostachys haplostachya*, which is part of an adaptive radiation of more than 50 species of Hawaiian mints (also including *Phyllostegia* and *Stenogyne*) derived from temperate North American *Stachys* [51,52]. The Hawaiian mints include numerous examples of derived viny and sub-shrub growth forms (e.g., [53]), and *H. haplostachya* and other Hawaiian mints are noted for their lack of leaf scent and essential oils [54]. More generally, the species native to the Hawaiian Islands produce fewer mono- and sesquiterpenes than species recently introduced there [55], suggesting that reductions in terpene production may be common in island plants. The convergent adaptations of increased height and woodiness and reduced terpenes in California *Stachys* and these Hawaiian mint species point to reduced vertebrate herbivory as a selective force shaping these traits. 

It may at first seem counterintuitive that island *S. bullata* genotypes outperformed their mainland relatives when grown in a mainland common garden (Figure 5A). However, it is important to note that our experimental setup precluded herbivory by mainland deer and gophers (see fencing and cages in Figure 3D), which may have disproportionately benefited island plants. Furthermore, common garden plants were exposed to relatively mesic conditions that may have favored island genotypes; in 2016, plants received supplemental water during their growing season, and the 2017 water year at the Santa Barbara Botanic Garden featured 118% of average annual precipitation (2017 = 63.3 cm, average = 53.4 cm).

While our results are consistent with the absence of mammalian herbivores as an adaptive explanation for reductions in plant defense traits, it is still possible that some of the observed differences may be attributable to divergent climatic conditions—particularly reduced temperature seasonality (Figure S5)—on the Channel Islands relative to the southern California mainland. This is especially the case for leaf area and SLA, which are not defenses per se and are expected to differ along climatic gradients, with greater leaf area and SLA when temperature seasonality is low (e.g., [56]). García-Verdugo et al. [57] also found increased leaf area for *Periploca laevigata* (Apocynaceae) on Macaronesian islands and attributed these differences to reduced temperature seasonality. By contrast, it is less clear why reduced temperature seasonality on islands would be associated with reductions in marginal leaf spines, cyanogenic glycosides, and leaf surface terpenes. 

In conclusion, we found evidence for reduced defensive traits in both chaparral shrubs and a perennial herbaceous plant from the California Channel Islands. Our results conflict with recent meta-analyses, which find limited evidence for reduced defenses being part of the island syndrome. We suggest that reduced defense against mammalian herbivores in island flora is likely to be a general phenomenon but that methodological drawbacks often obscure the detection of this pattern. Future research comparing island and mainland plant defense traits would benefit from a number of approaches (also highlighted more broadly in [26]). First, studies that focus on traits with unambiguous roles in plant defense against herbivores (e.g., latex exudation, alkaloids, cardenolides) may be more insightful for understanding selection imposed by herbivores than studies that focus on traits with multiple potential functions (e.g., phenolic compounds and leaf area). Second, comparisons that involve broad sampling from multiple island and mainland locations and from a wide range of taxa chosen without a priori knowledge of putative defense trait expression will provide the most robust inferences as to the degree of island/mainland divergence. Third, contrasts should focus on island groups whose floras have evolved in the absence of particular herbivore guilds (e.g., large browsing animals) if the goal is to determine the role of herbivores per se in driving defense trait evolution. Finally, for most taxa from the Channel Islands, there is no clear phylogenetic hypothesis for the degree of relatedness between island and mainland populations, including estimates of divergence times and contemporary gene flow, and future studies would greatly benefit from exploring the evolutionary history of island and mainland relatives. 

## 4. Methods

### 4.1. Background–California Channel Islands

The California Channel Islands are a group of uplifted volcanic oceanic islands off the coast of southern California that arose over the past 2–3 million years [58], ranging in size from 2.6 km^2^ (Santa Barbara Island) to 249 km^2^ (Santa Cruz Island) in land area (Figure 1A). The northern Channel Islands (including Santa Cruz and Santa Rosa) were periodically connected as a single landmass (Santa Rosae) during the Pleistocene Ice Ages [59], with as little as 10 km of separation between the island and mainland. The southern Channel Islands (including Santa Catalina) are generally more isolated from each other and the California mainland. The Channel Islands flora has a high degree of endemism and features many examples of insular woodiness and island gigantism [34,60].

Large mammalian herbivores have historically been absent from the California Channel Islands—with the notable exception of the pygmy mammoth (*Mammathus exilis*) [61]—but cattle, sheep, and pigs were introduced in the 1800s. In the last 50 years, concerted eradication efforts have removed large mammals from Santa Cruz and Santa Rosa Island; introduced mule deer (*Odocoileus hemionus*) and American bison (*Bison bison*) are still present on Santa Catalina Island. The Channel Islands also lack gophers, squirrels, and other burrowing mammals that are present on the mainland. 

### 4.2. Study 1: Chaparral Shrub Sampling

We selected five pairs of taxa characteristic of the chaparral plant community that occur on both the California Channel Islands and the nearby southern California mainland. Pairs were chosen because they are common representatives of the chaparral flora and also to match the taxa sampled in Bowen and Van Vuren (1997) [14]. Sampling consisted of either congeners or conspecifics (Figure 1B) from three plant families: Rosaceae (*Cercocarpus*, *Prunus*, *Heteromeles*), Papaveraceae (*Dendromecon*), and Rhamnaceae (*Ceanothus*). We collected leaf tissue in February and March of 2016 for use in morphological and chemical analysis. In total, we sampled 291 individual plants from five taxonomic pairs across six sites (three island, three mainland), for an average of approximately 10 plants per site (Figure 1B). 

We collected leaf tissue for morphological analysis from focal plants by clipping branches containing variable numbers of leaves. When possible, we collected a branch from both the lower (<1 m in height) and the upper (>2 m in height) portion of the plant canopy to capture morphological differences associated with accessibility to mammalian herbivores. For analysis of cyanogenic glycosides, we collected individual leaves from the lower portion of the plant canopy for three species (*Heteromeles*, *Prunus*, *Cercocarpus*) and, when possible, included both fully mature/expanded leaf tissue as well as young/actively expanding leaf tissue. Leaf chemistry samples were immediately frozen on dry ice and were later transferred to a −80 °C freezer until processing. For each sampled plant, we recorded its GPS coordinates (see Figure S1), elevation, and slope aspect (when relevant) using a handheld Garmin GPS device, and we also recorded the approximate stem diameter at 0.25 m above the ground using a digital caliper.

For each sampled branch, leaves were removed and imaged using a flatbed scanner (CanoScan LiDE 120, 2400 × 4800 dpi^2^) with a scalebar. We recorded the following measurements from each leaf: leaf area (without petiole), marginal leaf spinescence, and percent of leaf tissue missing due to herbivory. All measurements were taken using ImageJ v. 1.51 [62]. For a visual depiction of our measurement protocol, see Figure S2. Non-fully expanded leaves (*n* = 809) were measured but were excluded from subsequent analyses. We also measured specific leaf area (SLA) at the level of branches by taking the cumulative area of all fully expanded leaves (in cm^2^) and dividing this by their cumulative mass (in g). 

To measure cyanogenic glycoside (CNglc) content, we followed a modified version of the evolved hydrogen cyanide (HCN) protocol described in Experiment 2 of Gleadow et al. (2011). We only collected tissue for species in the Rosaceae (*Cercocarpus*, *Heteromeles*, *Prunus*), which are known to produce CNglcs, and included paired samples of mature (“old”) and expanding (“young”) leaf tissue from each plant, where possible. For a full description of methods used to quantify CNglc content, see Supplemental Materials. In total, we generated 194 measurements of CNglc content from 108 individual plants. 

We also sampled leaf tissue from two botanical gardens (Santa Barbara Botanic Garden and Rancho Santa Ana Botanic Garden) on the mainland that featured island and mainland genotypes of the species of interest (Figure 1B), grown from either seed or cuttings. All leaf tissue collection, morphological analysis, and chemical analysis were conducted in the same way as described above, although SLA was not measured for botanical garden plants. In total, we sampled an additional 40 plants (18 island and 22 mainland genotypes) from these common environments (Figure 1B). 

We also took advantage of a series of herbivore exclosures on Santa Catalina Island (see [63,64])—which still has introduced deer and bison present—to test for the potential effects of herbivore-mediated plasticity in plant traits. Because of the relatively small number of intact exclosures available, our sampling across species was somewhat uneven, though we were able to sample a total of 24 plants inside of exclosures and 35 plants outside of exclosures (Table S1). Note that plants in the herbivore exclosures experience gene flow from genotypes outside the exclosure through pollen and seed. 

### 4.3. Study 1: Abiotic Variation between Sites

Island and mainland sites have generally similar climates, although island locations may have more frequent nocturnal fog that reduces summertime evaporative water loss [65,66]. To formally measure climatic differences between island and mainland locations, we used recorded coordinates from each plant to extract bioclimatic variables from the WorldClim2 database at 1 km resolution [67] (Figure S3). We used principal component analysis (PCA) to explore variation in climate data and found that the first PC axis explained more than 83% of overall variation (Figure S4) and was dominated by a single bioclimatic variable, temperature seasonality (BIO4) (Figure S5). This axis separated island sites from the two more inland mainland sites (Stunt Ranch, Santa Monica Mtns.), which have higher temperature seasonality, while the third mainland site (Gaviota) had lower temperature seasonality and was more akin to island sites. The second PC axis explained 13% of the overall variation and included loadings for precipitation-related variables; this axis separated the drier Santa Catalina Island from all remaining sites (Figure S4). Island and mainland sites may also differ in soil properties; however, we did not attempt to quantify this potential variation.

### 4.4. Study 1: Statistical Analyses

We analyzed our data using multilevel linear mixed models implemented in the lme4 package [68] in R version 4.1.3 [69] to account for the hierarchical nature of our data. Response variables of interest were leaf area, SLA, marginal leaf spinescence, and leaf CNglc content. Leaf area and CNglc content were log-transformed to ensure that model-estimated confidence intervals were above 0; SLA and marginal leaf spinescence were untransformed. For marginal leaf spinescence, we only included *Heteromeles* and *Prunus* since these were the only species with stiff, rigid spines (Figure 1C). Likewise, because CNglc levels in *Cercocarpus* were ~100× lower than in *Prunus* and *Heteromeles* (and often below our detection limit), CNglc analysis was restricted to the latter two species. Covariates that were included in each model included the site of collection, canopy position (upper versus lower), north/south slope aspect, and east/west slope aspect. We considered including elevation and stem diameter (as a proxy for plant age) as covariates, though because of limited within-site and within-species variation in these measures, we ultimately omitted them from analyses. Furthermore, we attempted to include bioclimatic variables as covariates in these models, but because of the relatively limited spatial scale of sampling across sites (Figure 1A) and within sites (Figure S1) and the 1 km^2^ resolution of the bioclim dataset, we captured relatively little overall climatic variability for most bioclimatic variables (Figures S3 and S6).

For each response variable, we fit an overall model that included all samples collected in situ across all species (*n* = 4096 leaves from 291 plants). These models were of the form (in lme4 syntax):

Response variable ~ IM × Species + Covariates + (1|Site/Plant.ID/Branch.ID)

where IM corresponds to whether samples came from an island or mainland site. Plant species interacts with island vs. mainland status to allow for variation in the magnitude of island vs. mainland contrasts across species. The collection site was included as a random intercept, with plant ID nested within the site and branch ID nested within the plant ID. Since specific leaf area was calculated by pooling leaves from within branches, the SLA model does not include a branch ID term. To assess within-species differences in trait expression between islands and mainland locations, we used the emmeans package [70]. 

For two of the response variables (marginal spinescence, CNglc content), we included additional parameters based on a priori hypotheses. In the model considering marginal spinescence, we included an interaction between island/mainland status and canopy position to allow for the degree of spinescence heteroblasty to vary across locations (e.g., [16]). In the model considering CNglc content, we included a term for leaf age (old vs. young) based on our sampling scheme and predictions from optimal plant defense theory that younger leaf tissue should be more heavily defended against herbivores (e.g., [2]).

To test for genetically based differences in trait values, we analyzed samples collected from botanic garden plants in a separate set of linear mixed models. These models were similar to those described above and were of the form:

Response variable ~ Source_IM × Species + Covariates + (1|Plant.ID/Branch.ID)(1)

where *Source_IM* refers to whether each plants’ original provenance was an island or mainland location. As above, we also estimated within-species differences between island and mainland locations using the emmeans package [70].

Because we sampled the same species as Bowen and Van Vuren (1997) [14], we can compare insularity effects across studies (our field sampling, our botanic garden sampling, and the field sampling from Bowen and Van Vuren [14]). To do so, we calculated standardized effect sizes (Cohen’s *d_s_*) for each measured trait. Since Bowen and Van Vuren [14] only report t statistics and sample sizes, we used the following formula for Cohen’s *d_s_*:

(2) $$d_{s}=t\sqrt{\frac{1}{n_{1}}+\frac{1}{n_{2}}}$$

where *t* corresponds to the mean of their reported *t* statistics, and *n*_1_ and *n*_2_ correspond to sample sizes from island and mainland locations. To generate effect size estimates and corresponding confidence intervals from our in situ and botanic garden sampling, we used the effectsize package [71].

Finally, to test for the effects of introduced herbivores on plant traits on Santa Catalina Island, we separately analyzed all trait data from Santa Catalina and included a term to account for whether samples came from inside versus outside of an herbivore exclosure. 

### 4.5. Study 2: Stachys bullata–Background

*Stachys bullata* (Lamiaceae) is a perennial herbaceous plant that occurs in coastal California from approximately Orange County to the San Francisco Bay Area, with populations present on Santa Cruz, Santa Rosa, and Anacapa Islands. It reproduces both clonally via rhizomes and sexually and is described as being glandular, with aromatic foliage that is characteristic of many plants in the Lamiaceae. However, island populations have been noted to have non-aromatic foliage as well as larger leaves and flowers than their mainland relatives [34], and densities of glandular trichomes appear to be much lower on island plants (Figure 2C).

### 4.6. Study 2: Stachys bullata Common Garden Experiment

To determine whether observed trait variation between island and mainland *S. bullata* populations is environmentally or genetically determined, we set up a multi-year common garden experiment in which we grew island and mainland *S. bullata* genotypes together at the Santa Barbara Botanic Garden (SBBG). Plants were collected in the field in late 2015 from two island (Santa Cruz, Santa Rosa) and four mainland locations (Figure 2A) as rhizomes, which were transported to UC Davis and shallowly planted in potting mix. Plants were grown in 1-gallon pots for approximately three months and were then split into clonal replicates that were grown in their own 1-gallon pots. In total, we collected 44 *S. bullata* genotypes that were separated into 112 individual plants (Figure 2B).

In February 2016, we set up a common garden plot at the SBBG (Figure 2D) located on an east-facing slope that received partial or full sun throughout the year. Plants were spaced at a distance of 1 m apart from each other in a gridded pattern. The plot was surrounded by a 2 m fence to prevent browsing by deer, and each plant was enclosed in a cage made from hardware cloth to limit root herbivory by pocket gophers (*Thomomys bottae*), which were common at the site. We installed a drip irrigation system to assist with initial plant establishment. Plants were outplanted randomly with respect to island/mainland status in late February and early March of 2016 and received approximately 2L of water from a drip irrigation system at 1-week intervals between March and August 2016. In late August 2016, we ceased supplemental watering, and plants subsequently only received water from ambient precipitation. Plants became dormant in October 2016 and then subsequently began to regrow naturally in early February 2017. In addition, we set up a smaller common garden at the Santa Cruz Island Reserve, although due to concerns over the introduction of non-native genotypes, this common garden consisted of only genotypes from Santa Cruz Island.

We generated four categories of data from common garden *S. bullata* plants. Three measures (biomass, SLA, gas exchange) were related to plant growth, while one measure (leaf surface chemistry) was related to plant defense. For biomass measurements, we collected all above-ground biomass at the end of each growing season (in 2016 and 2017) and recorded its dry mass. For SLA, we collected leaf tissue from each plant in April 2017 and measured leaf area and dry mass from fully expanded *S. bullata* leaves. In April 2017, we used a Li-6800 portable photosynthesis system (LI-COR Biosciences Inc., Lincoln, NE, USA) to measure gas exchange rates on the most recent mature leaves that were sun exposed. For details on gas exchange measurements, see Supplemental Materials. 

For leaf chemistry, we focused on volatile organic compounds (VOCs) present on leaf surfaces and in glandular trichomes. Plants in the Lamiaceae are known for their exceptional diversity of terpenoid compounds [72], which are a diverse group of plant secondary metabolites thought to be involved in defense against herbivores and pathogens, plant communication, and modulating thermal and oxidative stress [73,74]. We used a modified version of the protocol described in Pratt et al. (2014) [75] for measuring terpenes in *Artemisia californica* (Asteraceae). Briefly, in April of 2017, after >1 year of growth in the common garden, we used a hole punch to collect six leaf discs, each from a different leaf, from approximately 75 *Stachys* plants across all genotypes. Leaf surface chemistry was quantified using gas chromatography-mass spectrometry (GCMS) for a subset of 47 of these plants (Figure 2B). For a full description of chemical methods, see Supplementary Materials.

### 4.7. Study 2: Stachys Data Analysis

We analyzed aboveground biomass using a linear mixed effects model of the form:

Aboveground biomass ~ IM + Year + (1|Source.Pop/Genotype) + (1|Column) + (1|Row)(3)

where IM refers to whether a given plant originated from an island or mainland site, and column and row refer to the location of plants within the common garden grid. To analyze gas exchange measurements, because of our smaller sample, we used a simple linear model with net carbon assimilation (A_net_) as the response and provenance (island vs. mainland) as the predictor.

To analyze plant chemistry, we divided each integrated peak area by its corresponding internal standard peak area to standardize all values. We added all peaks together to achieve a cumulative compound abundance measure and also separated compounds based on their biochemical pathway (e.g., monoterpenes, sesquiterpenes, aromatic compounds). We used non-metric multidimensional scaling to visualize compositional differences between sites.

## Figures and Tables

**Table 1:** Comparison of trait values for island and mainland plants sampled in situ, shown for each taxonomic pair. Mean values correspond to model-estimated marginal means, which are averaged across each of the three island and three mainland sampling sites. Positive t-statistics indicate positive insularity effects. Statistically significant differences (*p* < 0.05) are highlighted in bold.

Field Sampling					
Trait	Species Pair	Island Mean	Mainland Mean	t	*p*
Leaf area	***Ceanothus***	**1.99**	**1.07**	**5.058**	**<0.001**
	***Cercocarpus***	**4.06**	**2.91**	**2.558**	**0.018**
	***Dendromecon***	**5.24**	**2.37**	**6.123**	**<0.001**
	*Heteromeles*	8.67	7.06	1.793	0.095
	***Prunus***	**11.51**	**4.58**	**7.817**	**<0.001**
Specific leaf area (SLA)	***Ceanothus***	**4.76**	**3.11**	**2.554**	**0.015**
	*Cercocarpus*	7.84	7.17	1.064	0.296
	*Dendromecon*	7.18	7.50	−0.485	0.631
	*Heteromeles*	4.59	4.15	0.791	0.439
	***Prunus***	**8.29**	**6.30**	**3.403**	**0.002**
Marginal leaf spinescence	*Heteromeles*	1.86	3.54	−2.406	0.060
	***Prunus***	**1.79**	**7.73**	**−8.484**	**<0.001**
Cyanogenic glycoside content	***Heteromeles***	**0.48**	**1.20**	**−2.635**	**0.037**
	*Prunus*	1.55	2.91	−1.796	0.122

**Table 2:** Comparison of trait values for island and mainland plants sampled from botanic gardens, shown for each taxonomic pair. Mean values correspond to model-estimated marginal means. Positive t-statistics indicate positive insularity effects. Statistically significant differences (*p* < 0.05) are highlighted in bold. Note that SLA was not measured for botanic garden plants.

Botanic Garden Sampling					
Trait	Species Pair	Island Mean	Mainland Mean	t	*p*
Leaf Area	*Ceanothus*	1.46	0.98	0.992	0.331
	*Cercocarpus*	2.63	2.32	0.327	0.746
	*Dendromecon*	6.10	7.93	−0.562	0.579
	*Heteromeles*	11.23	8.61	0.550	0.587
	***Prunus***	**19.92**	**5.72**	**3.616**	**0.001**
Marginal leaf spinescence	*Heteromeles*	1.35	1.68	−0.305	0.766
	***Prunus***	**0.00**	**3.17**	**−10.885**	**<0.001**
Cyanogenic glycoside content	*Heteromeles*	0.42	0.93	−1.841	0.085
	*Prunus*	1.34	3.48	−2.000	0.058

## Data Availability

All raw data and code are available on Github at the following link: https://github.com/micahfreedman/manuscripts/tree/master/Island_Mainland, accessed on 10 March 2024. Data and scripts used for analysis are also available through Dryad: https://doi.org/10.5061/dryad.51c59zwgj, accessed on 10 March 2024.

## Supplementary Materials

The following supporting information can be downloaded at: https://www.mdpi.com/article/10.3390/plants13071026/s1, Figure S1: Maps of sampling locations for chaparral shrubs across each of the six sampling sites. Figure S2: Depiction of sampling scheme for chaparral shrub trait analysis. Figure S3: Maps depicting variation in each of the 19 recorded bioclimatic variables. Figure S4: Principal component analysis of climatic variation between sampling sites. Figure S5: PCA biplot showing contributions of each climate variable to overall loadings. Figure S6: Histograms showing the number of observations falling into each bioclimatic variable bin for all 291 field-sampled plants. Figure S7: Boxplots showing distribution of plant-level mean values from field sampling for leaf area (A), specific leaf area (B), marginal leaf spinescence (C), and cyanogenic glycoside content (D). Figure S8: Trait values for each species across island and mainland locations, based on common garden sampling. Figure S9: Depiction of spinescence heteroblasty, with spinescence shown based on canopy position for island and mainland species of interest. Figure S10: Cyanogenic glycoside (CNglc) content in leaf tissue, separated based on species, island/mainland status, and leaf tissue age. Figure S11: Ordination of *S. bullata* leaf chemical profiles for genotypes from Santa Cruz Island grown in two common gardens: SBBG (Santa Barbara Botanic Garden) and SCI (Santa Cruz Island field station). Figure S12: Example of differences in growth form between common garden island and mainland *S. bullata*. Figure S13: Standard calibration curve for CNglc detection method. Table S1: Counts of plants sampled from Santa Catalina Island, separated based on whether they were located inside versus outside of deer exclosures built by the Catalina Island Conservancy. Table S2: Summary of models testing for an effect of deer exclosures on trait expression on Santa Catalina Island. Supplementary Methods, which contains details of cyanogenic glycoside measurement, leaf VOC collection and measurement, statistical analysis of GC-MS data, and LI-COR instrument operation.
