# Supplementary material for: Evidence for Reductions in Physical and Chemical Plant Defense Traits in Island Flora
Source: Plants (Basel). 2024 Apr 3;13(7):1026. doi: 10.3390/plants13071026 (PMC11013342; doi:10.3390/plants13071026)
Supplement: Supplementary file 1 [file plants-13-01026-s001.zip › Supplementary Figures.pdf]

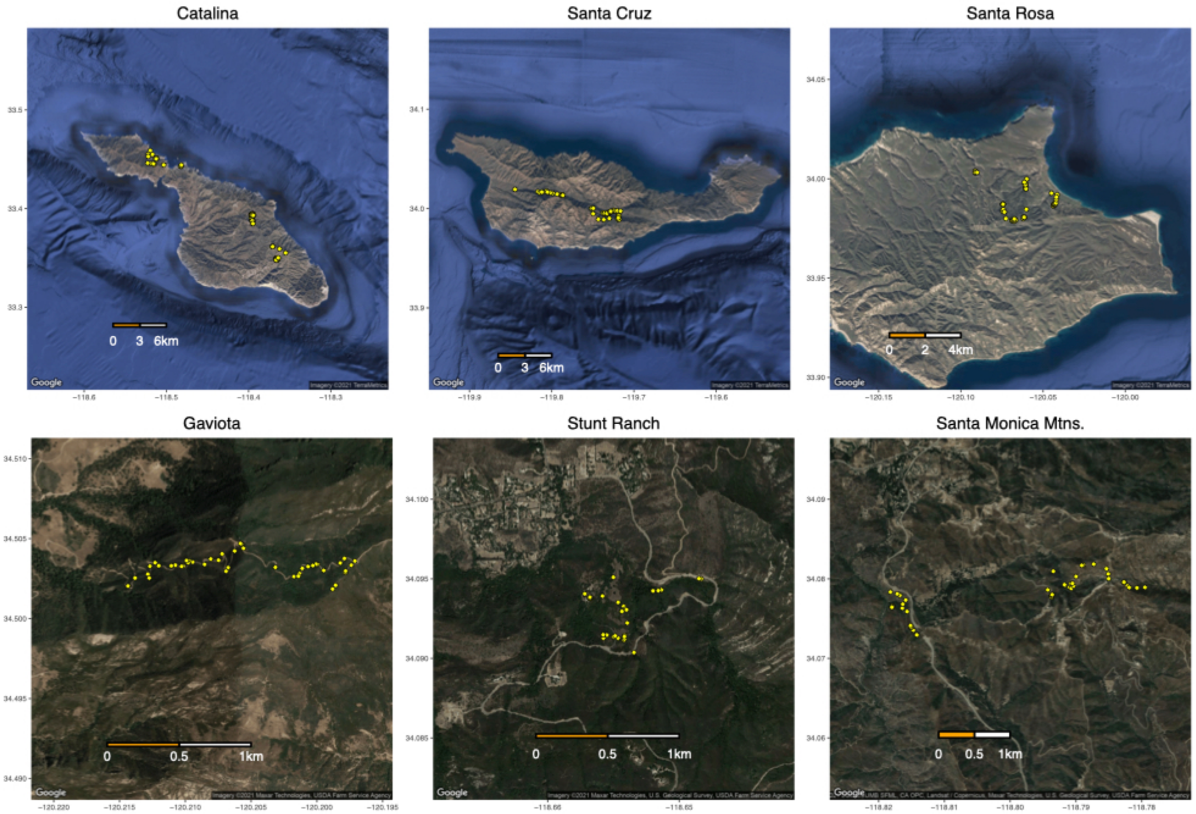

Figure S1 – Maps of sampling locations for chaparral shrubs across each of the six sampling sites. Each point corresponds to a single plant. Maps were generated using the ggmap package (Kahle and Wickham 2013) and the Google Earth Engine API.

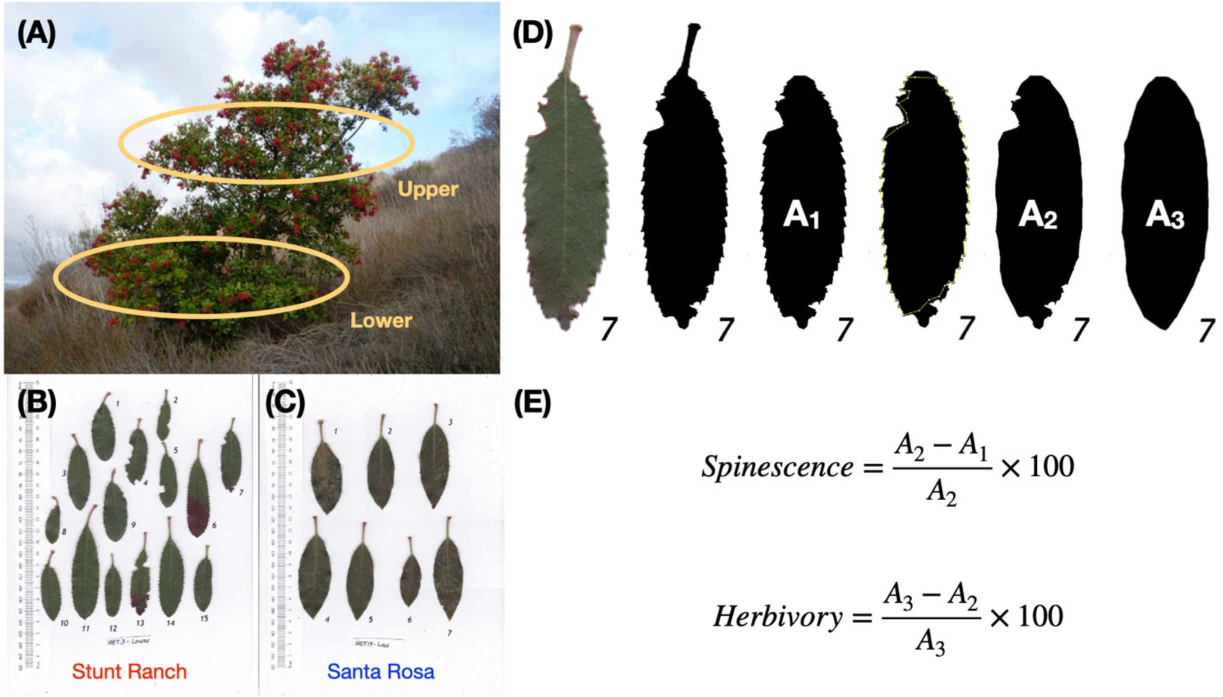

Figure S2 – Depiction of sampling scheme for chaparral shrub trait analysis. **(A)** When possible, branches were collected from both the upper and lower canopy of focal shrub species. **(B)** Example of scanned leaves from the lower canopy of Heteromeles individual #3, sampled from Stunt Ranch (mainland). **(C)** Scanned leaves from Heteromeles individual #19, from Santa Rosa Island. **(D)** For each individual leaf (here leaf number 7 from Panel B is shown), we used ImageJ to measure its morphology. After converting each image into black/white pixels, we recorded the leaf area minus the petiole (denoted as A_1). Spinescence was measured by connecting the vertices of marginal spines using the polygon selection, then filling in the resulting object. The area of this object (A_2) was then used to determine the spinescence percentage, shown in **(E)**. Herbivory was recorded in a similar manner. Spinescence was coded as 'NA' for samples with herbivory levels > 10% of leaf area removed, and leaves that were determined to be not fully expanded were excluded from analysis. Specific leaf area was determined by summing the cumulative area of each fully expanded leaf (in cm^2) and dividing this by the cumulative mass of those leaves (in g).

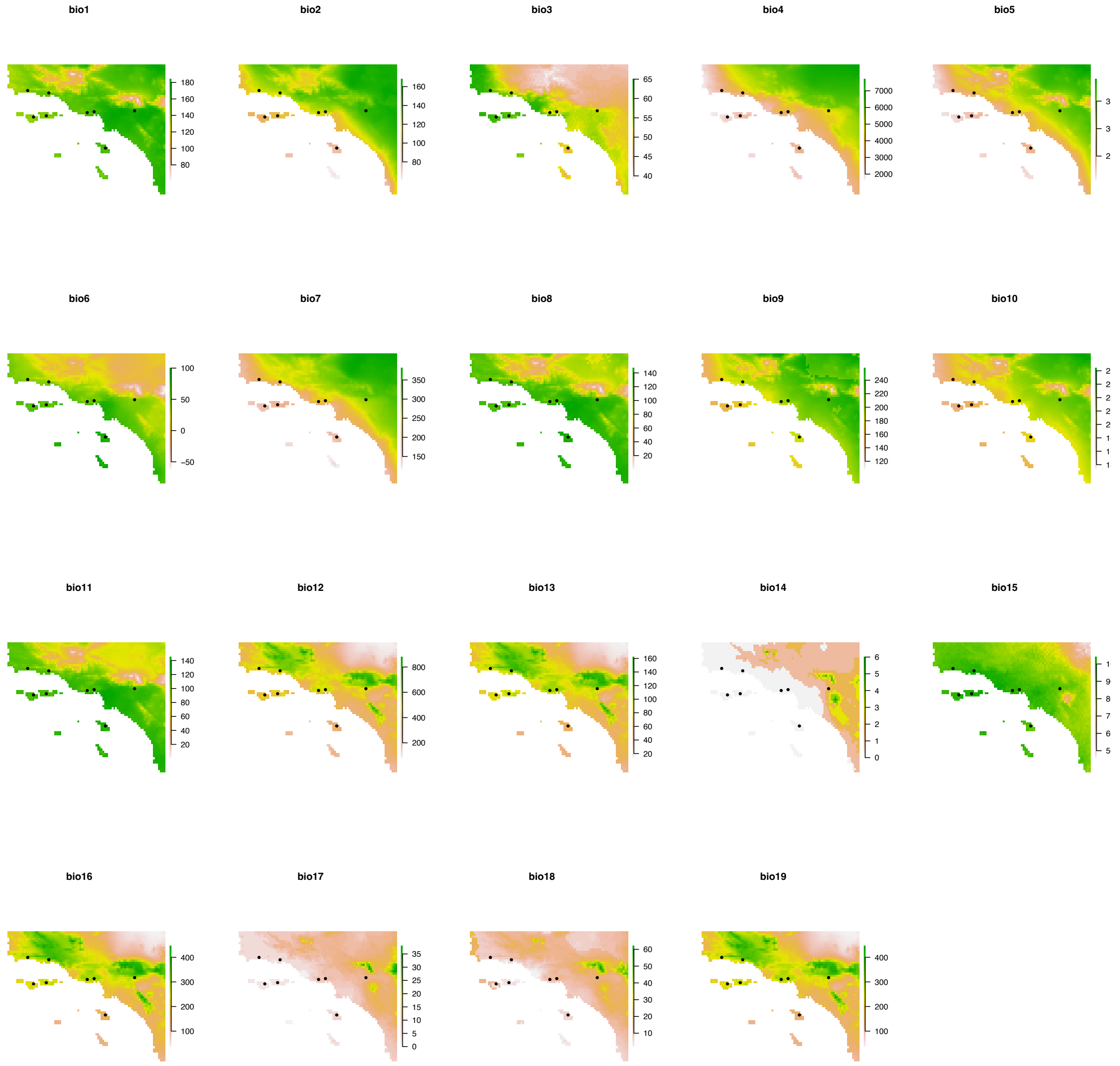

Figure S3 – Maps depicting variation in each of the 19 recorded bioclimatic variables. Sampling loations (both field and common garden) are shown as black points. **BIO1** = Annual Mean Temperature, **BIO2** = Mean Diurnal Range (Mean of monthly (max temp - min temp)), **BIO3** = Isothermality (BIO2/BIO7) (×100), **BIO4** = Temperature Seasonality (standard deviation ×100), **BIO5** = Max Temperature of Warmest Month, **BIO6** = Min Temperature of Coldest Month, **BIO7** = Temperature Annual Range (BIO5-BIO6), **BIO8** = Mean Temperature of Wettest Quarter, **BIO9** = Mean Temperature of Driest Quarter, **BIO10** = Mean Temperature of Warmest Quarter, **BIO11** = Mean Temperature of Coldest Quarter, **BIO12** = Annual Precipitation, **BIO13** = Precipitation of Wettest Month, **BIO14** = Precipitation of Driest Month, **BIO15** = Precipitation Seasonality (Coefficient of Variation), **BIO16** = Precipitation of Wettest Quarter, **BIO17** = Precipitation of Driest Quarter, **BIO18** = Precipitation of Warmest Quarter, **BIO19** = Precipitation of Coldest Quarter.

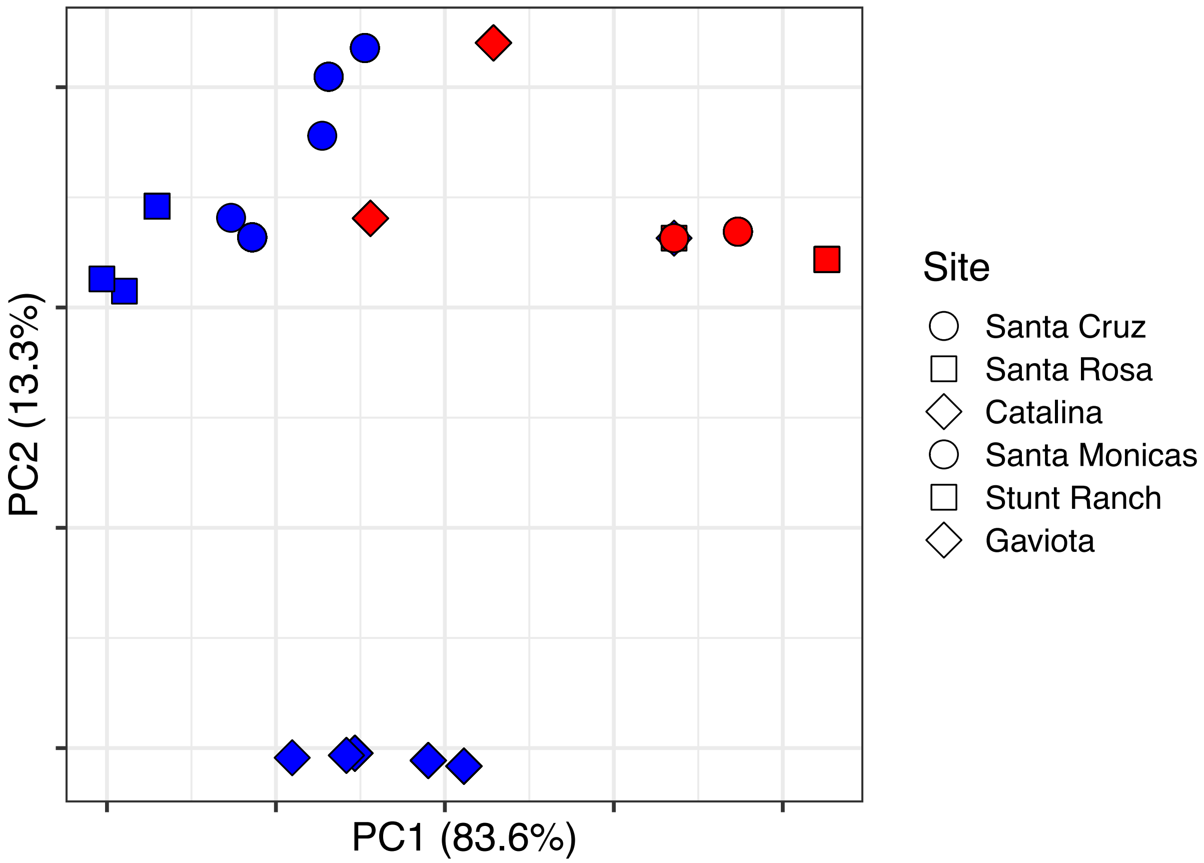

Figure S4 – Principal component analysis of climatic variation between sampling sites, with islands shown in blue and mainland locations shown in red. Each point corresponds a single 1 km^2 cell, each of which contains multiple sampled plants. Island and mainland locations were differentiated along PC axis 1, which explained 83.6% of overall variation and was dominated by loading corresponding to bio4 (temperature seasonality) (see Figure S5 below). PC axis 2 separated Santa Catalina Island from all other locations and corresponded to precipitation related variables (e.g., bio12 = annual precipitation).

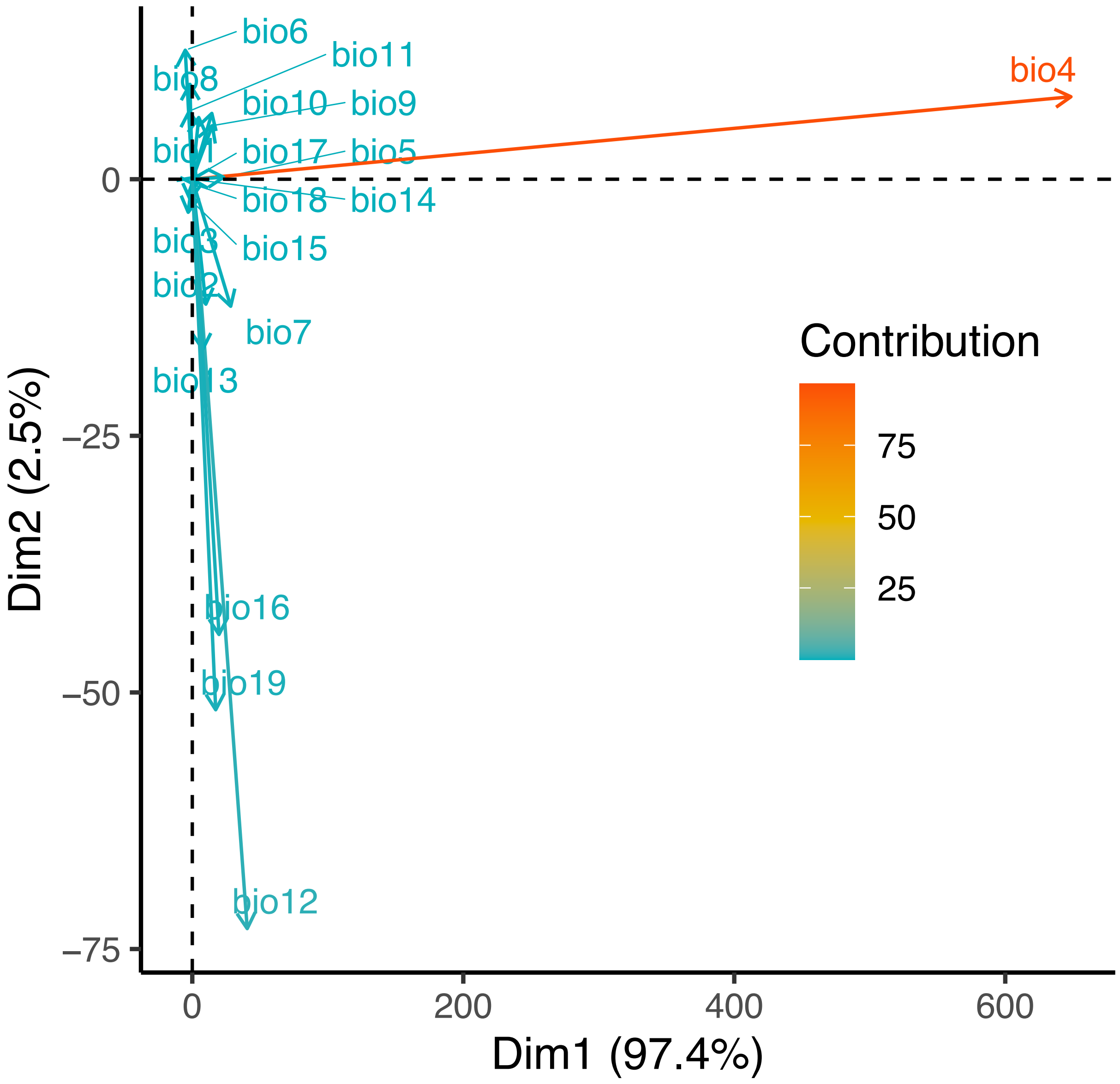

Figure S5 – PCA biplot showing contributions of each climate variable to overall loadings. Overall differentiation among sampling locations is dominated by a single bioclimatic variable (bio4 = temperature seasonality).

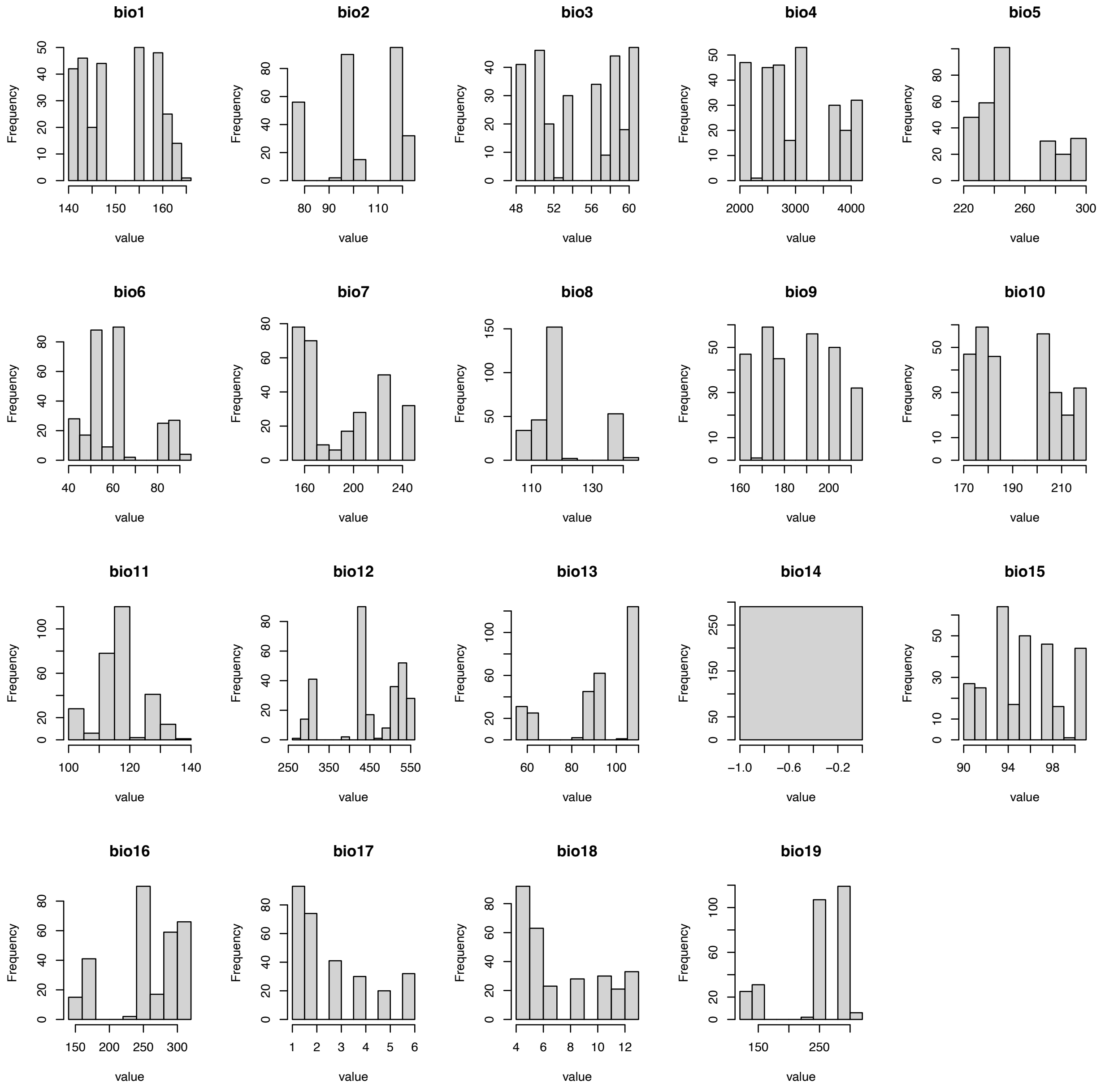

Figure S6 – Histograms showing the number of observations falling into each bioclimatic variable bin for all 291 field sampled plants. Note that for we captured relatively little overall variation in climate space because of the limited spatial extent of our sampling; hence, most bioclimatic variables include 10 or fewer grid cells with unique values. All sampled grid cells received values of 0 for bio14 (precipitation of driest month). **BIO1** = Annual Mean Temperature, **BIO2** = Mean Diurnal Range (Mean of monthly (max temp - min temp)), **BIO3** = Isothermality (BIO2/BIO7) (×100), **BIO4** = Temperature Seasonality (standard deviation ×100), **BIO5** = Max Temperature of Warmest Month, **BIO6** = Min Temperature of Coldest Month, **BIO7** = Temperature Annual Range (BIO5-BIO6), **BIO8** = Mean Temperature of Wettest Quarter, **BIO9** = Mean Temperature of Driest Quarter, **BIO10** = Mean Temperature of Warmest Quarter, **BIO11** = Mean Temperature of Coldest Quarter, **BIO12** = Annual Precipitation, **BIO13** = Precipitation of Wettest Month, **BIO14** = Precipitation of Driest Month, **BIO15** = Precipitation Seasonality (Coefficient of Variation), **BIO16** = Precipitation of Wettest Quarter, **BIO17** = Precipitation of Driest Quarter, **BIO18** = Precipitation of Warmest Quarter, **BIO19** = Precipitation of Coldest Quarter.

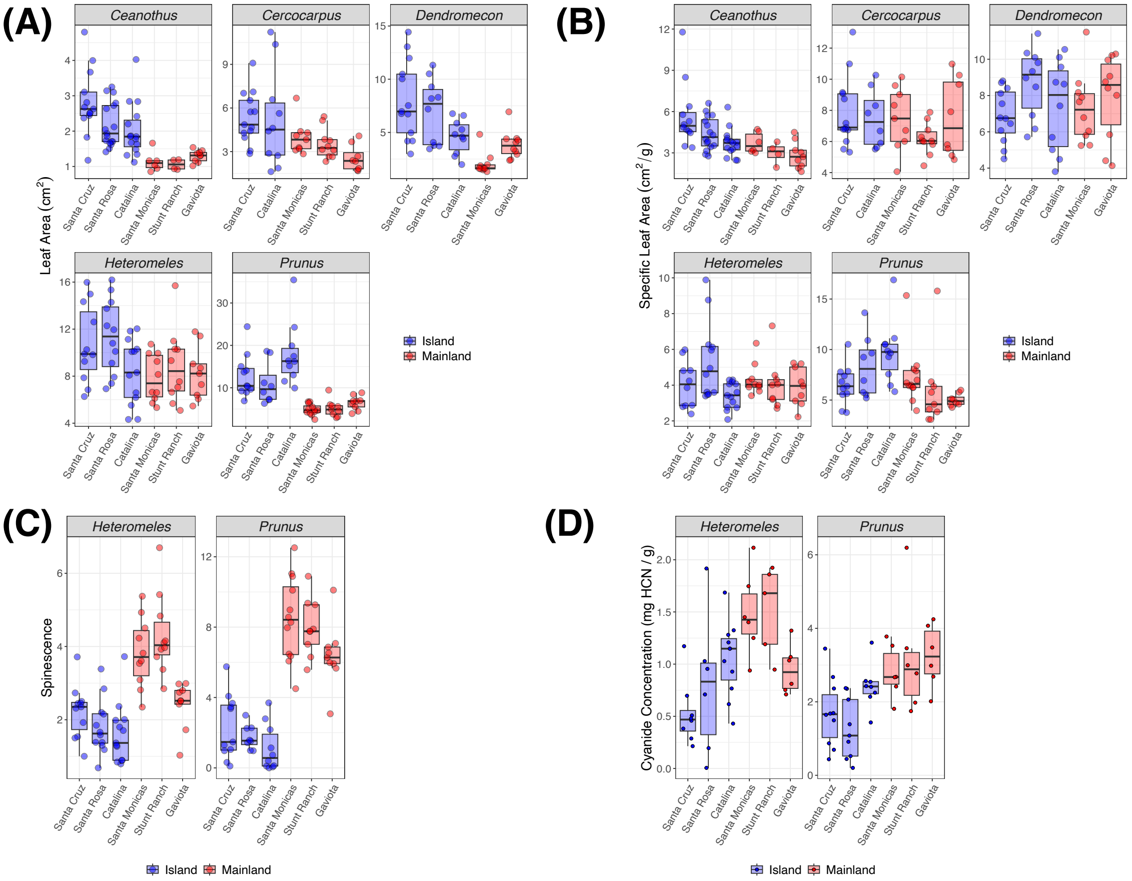

Figure S7 – Boxplots showing distribution of plant-level mean values from field sampling for leaf area (A), specific leaf area (B), marginal leaf spinescence (C), and cyanogenic glycoside content (D).

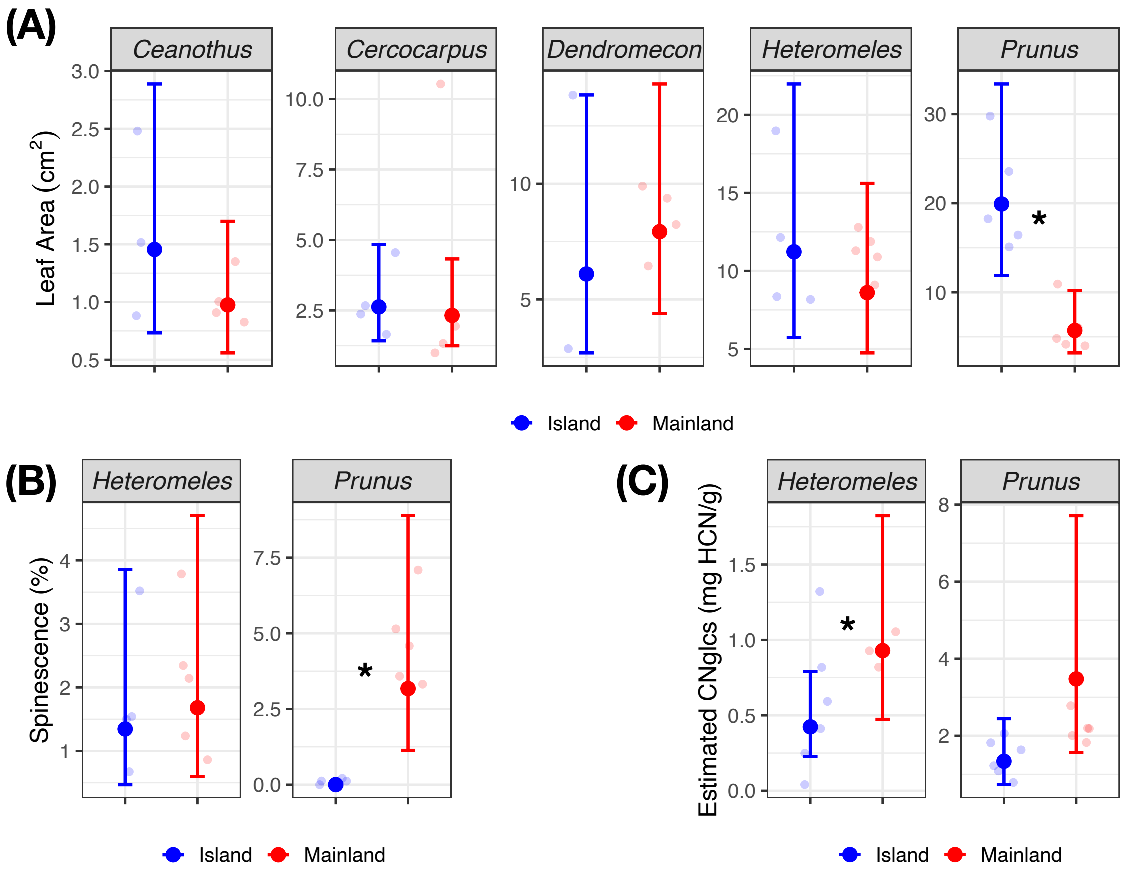

Figure S8 – Trait values for each species across island and mainland locations, based on common garden sampling. Model-estimated marginal means and 95% confidence intervals are shown with solid points and lines. Each pale dot corresponds to a single plant-level mean, which is itself the mean of leaf traits from branches in the upper and lower canopy. Measured leaf traits were (**A**) leaf area, (**B**) marginal leaf spinescence, and (**C**) concentrations of cyanogenic glycosides (*Heteromeles* and *Prunus* only for B and C). Asterisks correspond to significant ($p < 0.05$) differences between island and mainland plants within each species *x* trait combination.

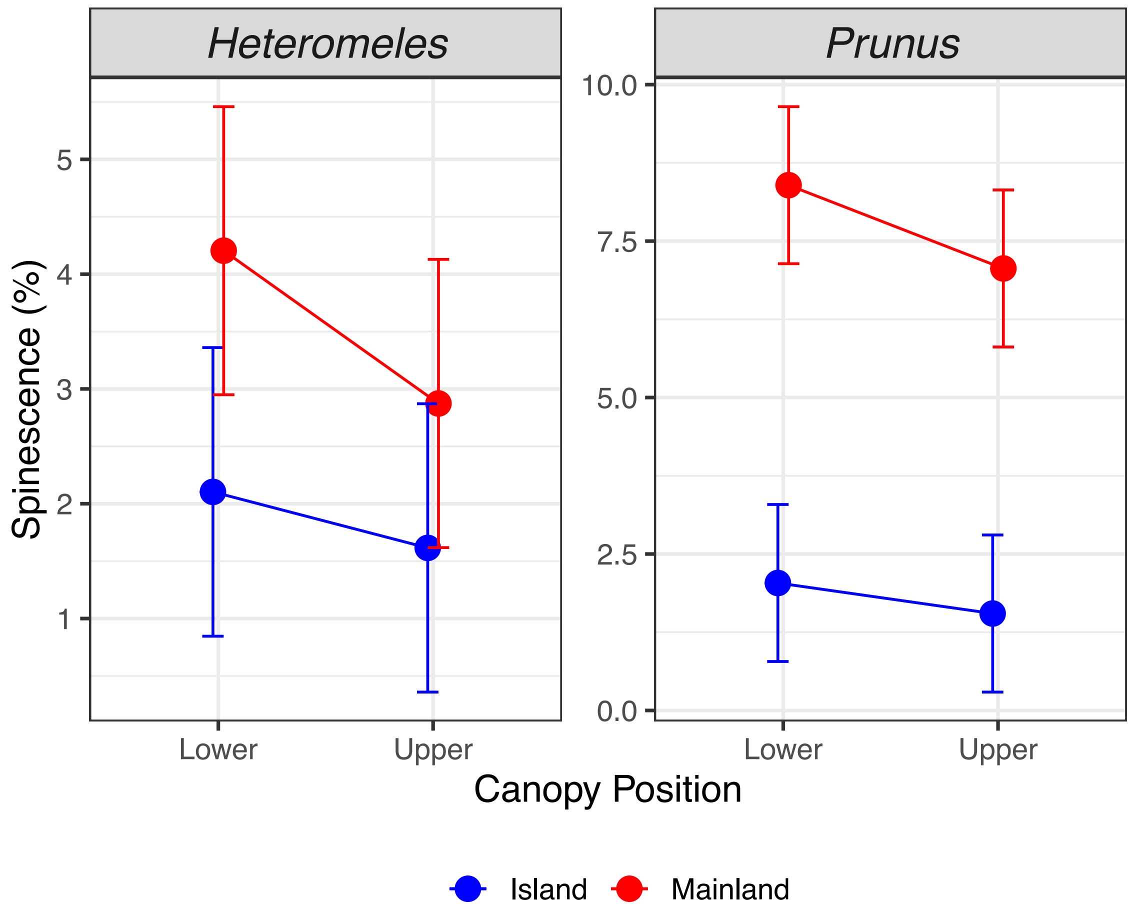

Figure S9 – Spinescence heteroblasty was more pronounced for mainland than island plants, especially for *Heteromeles*. Estimated marginal means and associated 95% confidence intervals are shown for the two species that had clear marginal spines. Across all contexts, marginal spines were more prominent from the lower than the upper canopy; this pattern is known as spinescence heteroblasty.

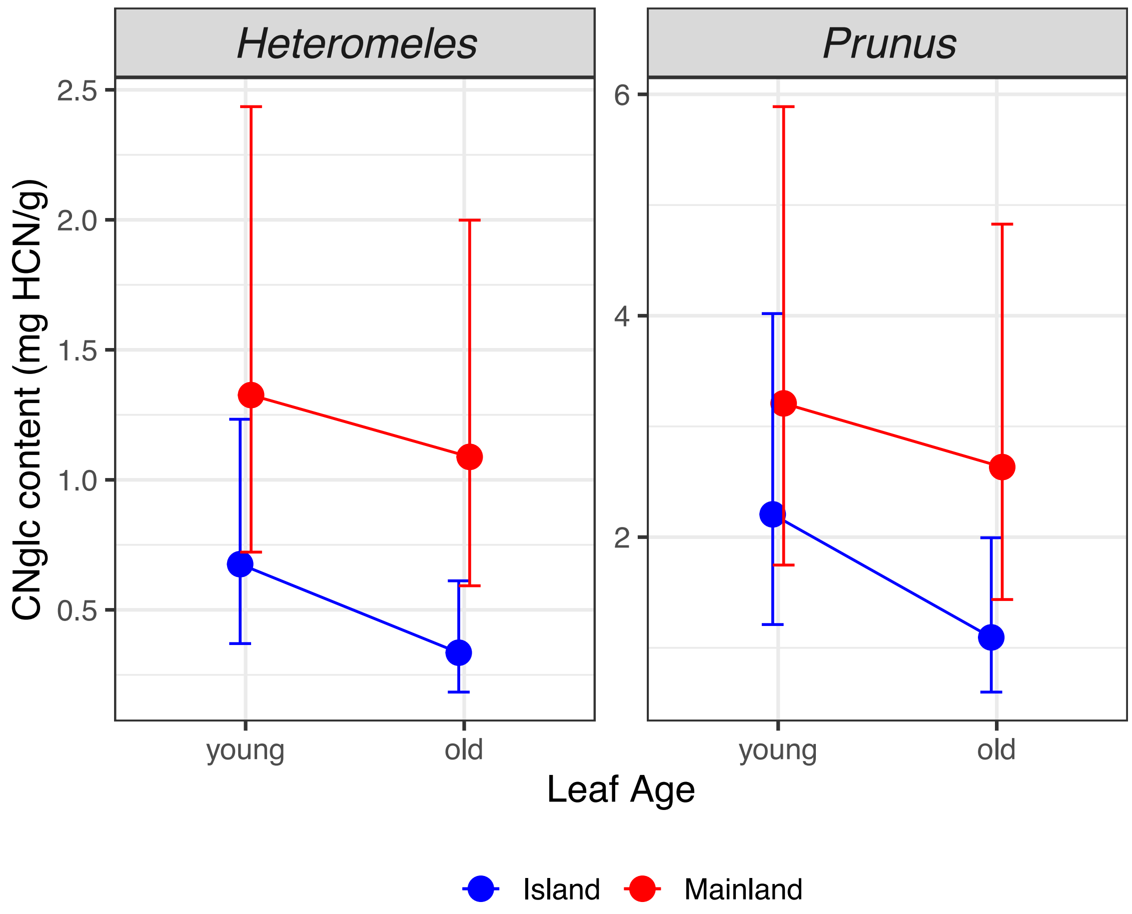

Figure S10 – Cyanogenic glycoside (CNglc) content, separated based on species, island/mainland status, and leaf tissue age. Across all contexts, leaf tissue that was younger and had not fully expanded contained higher concentrations of CNglcs, and mainland plants contained higher concentrations than island plants. However, island plants showed a more pronounced decline in CNglc content with age than mainland plants (IM status *x* age interaction: $t = 3.532$, $p < 0.001$).

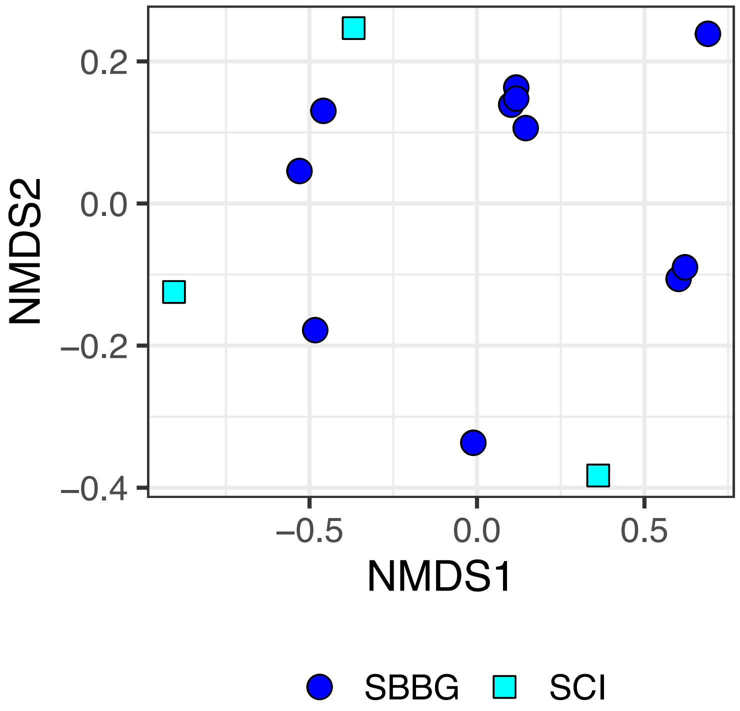

Figure S11 – Ordination of *S. bullata* leaf chemical profiles for genotypes from Santa Cruz Island grown in two common gardens: SBBG (Santa Barbara Botanic Garden) and SCI (Santa Cruz Island field station). Garden location did not affect leaf chemistry.

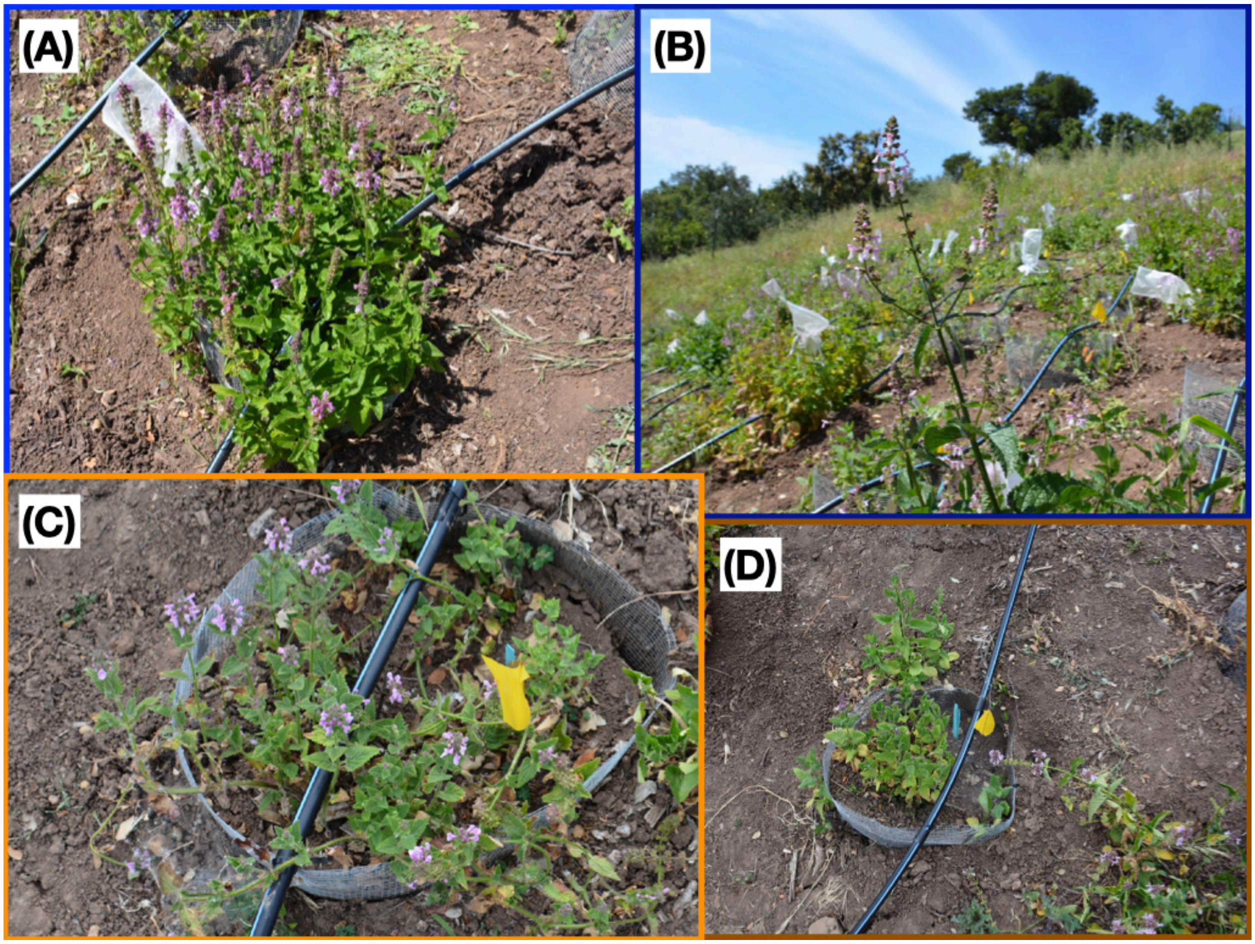

Figure S12 – Example of differences in growth form between common garden island and mainland *Stachys bullata*. **(A)** Plant from Santa Cruz Island, illustrative of the upright growth form and high above-ground biomass in island genotypes. **(B)** Plant from Santa Rosa Island demonstrating woody and branching upright stems. Santa Rosa *S. bullata* genotypes had white/light pink flower color (distinct from the pink flower color seen in all other populations). **(C)** Plant from a mainland location (El Capitan) demonstrating shorter stature, creeping growth habit, and "fuzzy" appearance typical of mainland genotypes. **(D)** Plant from another mainland location (Gaviota), with a stem from a Santa Cruz Island plant (at right) growing laterally into the frame.

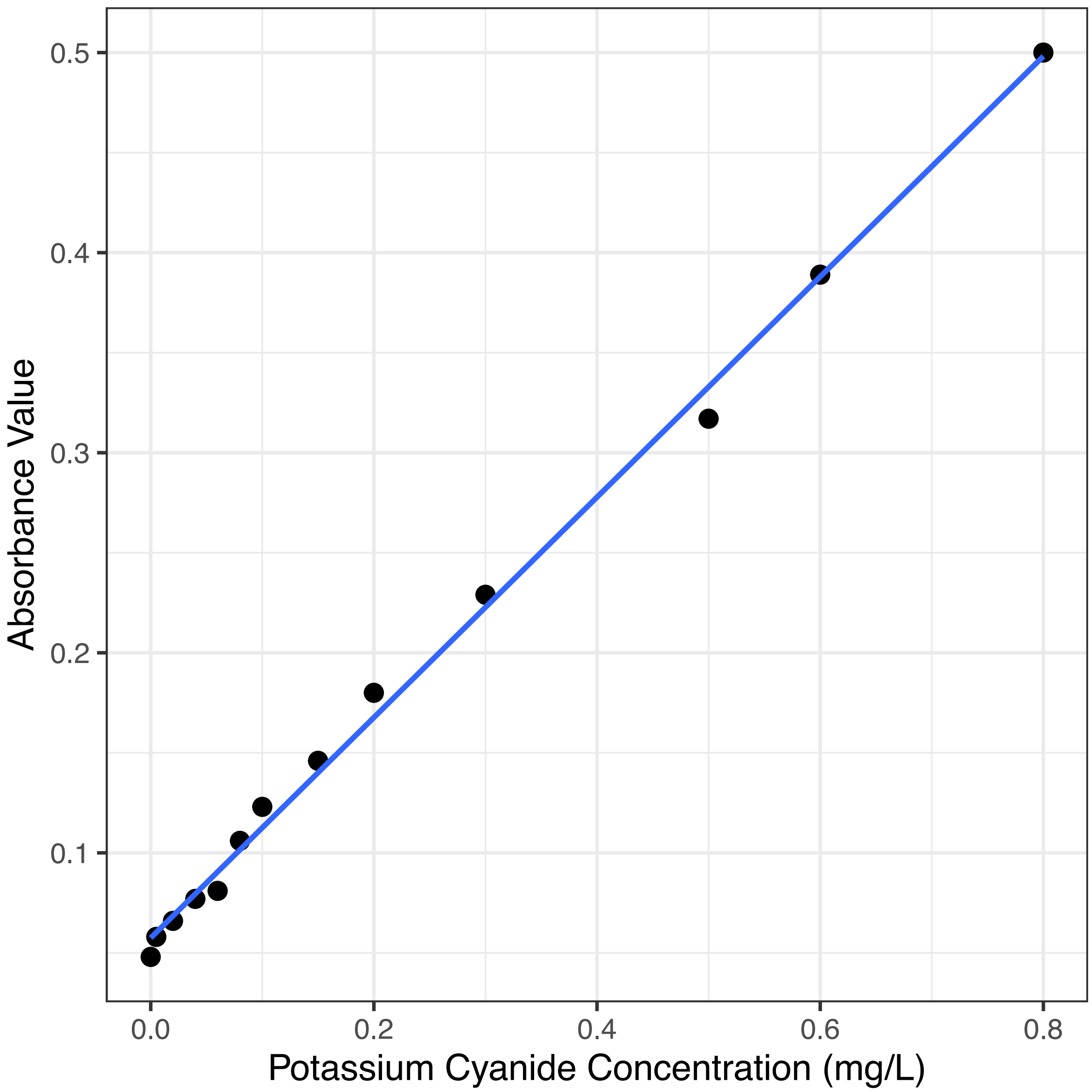

Figure S13 – Calibration curve for cyanogenic glycoside detection method. We prepared a dilution series using KCN (Sigma Aldrich) and then used our detection kits according to the package instructions to quantify cyanide content. Note that although the test kits suggested that they were only intended for use between 0 – 0.200 mg/L of cyanide, absorbance values increased linearly up to 0.800 mg/L.

**Species**	**N Outside Exclosures**	**N Inside Exclosures**
Ceanothus	8	7
Cercocarpus	6	4
Dendromecon	4	6
Heteromeles	7	6
Prunus	10	1
**Total**	35	24

Table S1 – Counts of plants sampled from Santa Catalina Island, separated based on whether they were located inside versus outside of deer exclosures built by the Catalina Island Conservancy.

Trait	Exclosure effect	t	p
Leaf area	0.586	1.700	0.097
Specific leaf area	-1.165	-0.730	0.473
Spinescence	0.127	0.245	0.809
CNglc content	0.064	0.203	0.842

Table S2 – Summary of models testing for an effect of deer exclosures on trait expression on Santa Catalina Island. Exclosure effect refers to the model coefficient describing the difference in trait values for exclosure present vs. exclosure absent (i.e., a positive value indicates a positive effect of exclosures). The only trait that showed a potential impact of exclosures was leaf area, with marginally larger leaves on plants growing inside of deer exclosures. These results suggest that herbivore-induced plasticity is unlikely to be a major factor explaining observed differences between island and mainland plants.
