# Supplementary material for: Evidence for Reductions in Physical and Chemical Plant Defense Traits in Island Flora
Source: Plants (Basel). 2024 Apr 3;13(7):1026. doi: 10.3390/plants13071026 (PMC11013342; doi:10.3390/plants13071026)
Supplement: Supplementary file 1 [file plants-13-01026-s001.zip › Supplementary Methods.pdf]

Supplementary Methods

Cyanogenic glycoside measurement

Cyanogenic glycosides were measured from plant tissue collected in the field on dry ice and stored in a -80°C freezer prior to analysis. A piece of frozen leaf tissue was massed, transferred to a 1.5 mL tube with stainless steel grinding beads, added to liquid nitrogen, and pulverized in a tissue lyser (Qiagen TissueLyser LT) at 50 Hz for 30 seconds total. Next, we added 1 ml of 0.1M citrate buffer to each tube containing ground frozen tissue, which was then transferred into a larger 15 mL tube containing a separate 1.5 mL tube with 1 ml of 1M NaOH. This larger 15 mL tube was sealed, allowed to incubate at room temperature for 1 hour, and then transferred into a 37°C chamber to incubate for 12 hours overnight. The amount of NaCN captured in NaOH was measured via the pyridine-pyrazalone method using reagents purchased from Hanna Instruments (HI93714-01). The 1 mL of dissolved NaCN was added to 30 mL of deionized water and neutralized with 0.5 M acetic acid. We then added 1 mL of this solution to a separate container of 10 mL of deionized water and added reagents A, B, and C according to manufacturer instructions. The resulting color change (from clear to blue) was measured in a plastic cuvette inserted into a spectrometer (VWR V-1200), with absorbance was measured at 595 nm. This absorbance value was then compared to a calibration curve prepared using known concentrations of KCN (Figure S13) to obtain a sample concentration. We also experimentally added 0.5 mg of β-glucosidase (Sigma Aldrich) to a small subset (n = 3) of tissue samples in citrate buffer to test whether endogenous enzyme activity was sufficient to hydrolyze all cyanogenic glycosides; this addition did not appreciably change the resulting absorbance values.

Leaf VOC collection and measurement

Leaf discs of *Stachys bullata* were added to 2 mL glass vials containing 500 μL of dichloromethane and 5 μL of a 90 ng/ml tetralin internal standard and were stored at -20°C until processing. Vials were sonicated for 10 minutes, and then 200 μL of the eluent was filtered through a modified capillary tube containing powdered silica into a 250 μL GC vial insert. Samples were injected onto an Agilent 7890B gas chromatograph fitted with a 30 m × 0.25 mm × 0.25 um HP-5 Ultra Inert column coupled to an Agilent 5977A mass spectrometer (Agilent Technologies) using a 5:1 split ratio, a 1 μL injection volume, and an inlet temperature of 250°C. The initial oven temperature was 40°C, held for 3 minutes, followed by a temperature ramp of 5°C/min up to 210°C, followed by a subsequent ramp of 20°C/min to 300°C, followed by a final hold at 300°C for one minute. Helium was used as the carrier gas at a flow rate of 1.2 mL/min. Electron impact mass spectra were obtained by scanning between 30-550 *m/z*.

GC/MS data were processed using MassHunter GC/MS Acquisition software version B.07.00 and MSD ChemStation Enhanced Data Analysis Software version F.01.00 (Agilent). Peaks were initially called automatically using the RTE integrator and a detection threshold limit of 1.0% of the largest peak. Chromatograms were manually annotated to include peaks that were visible but fell below this detection threshold. Peak alignment was based on retention times, and we assigned identifications to compounds by comparing mass spectra and retention times to published databases (Adams 2007, NIST mass spectral library). In total, our dataset included 79 peaks.

Statistical analysis of GC-MS data

To visualize multivariate disparity among populations in chemical composition, we used non-metric multidimensional scaling (nMDS) implemented in the vegan package version 2.5-7 (Oksanen et al. 2020), specifying Bray-Curtis distances, k = 8 dimensions, and 1000 random starts in the metaMDS function; group-level differences between genotypes

from island versus mainland sites were assessed using perMANOVA implemented via the adonis2 function in vegan (Oksanen et al. 2020).

LI-COR operation

In April 2017, we measured gas exchange from a set of 26 *S. bullata* plants growing at the Santa Barbara Botanic Garden. We used the 2x3 cm chamber with the attached light source to provide uniform light levels. Measurements were completed between 10:00h and 12:00h and chamber conditions were set to imitate the ambient conditions, viz. 31 °C, 400 ppm CO_2, 1600 PAR. The samples were acclimated in the chamber for at least three minutes or until stability in ΔCO_2 and ΔH_2O was achieved (when coefficients of variation were <0.25%). Two measurements were logged at 30 s intervals and the mean value of those data were used in analyses.
